# Retrospective Analysis of Vitamin D Deficiency in an Adult Population of Arad County, Western Romania (2019–2022)

**DOI:** 10.3390/life14020274

**Published:** 2024-02-18

**Authors:** Daniela Teodora Marti, Alexandru Nesiu, Cornel Balta, Tudor Rares Olariu, Alin Gabriel Mihu, Anca Hermenean, Daniela Adriana Oatis

**Affiliations:** 1 Department of Biology and Life Sciences, Faculty of Medicine, Vasile Goldis Western University of Arad, 310025 Arad, Romania; dana_m73@yahoo.com (D.T.M.); alexnesiu@yahoo.com (A.N.); danielaoatis@gmail.com (D.A.O.); 2 “Aurel Ardelean” Institute of Life Sciences, Vasile Goldis Western University of Arad, 86 Rebreanu, 310414 Arad, Romania; baltacornel@gmail.com; 3 Discipline of Parasitology, Department of Infectious Disease, Victor Babes University of Medicine and Pharmacy, 300041 Timisoara, Romania; rolariu@umft.ro; 4 Center for Diagnosis and Study of Parasitic Diseases, Victor Babes University of Medicine and Pharmacy, 300041 Timisoara, Romania; 5 Clinical Laboratory, Municipal Clinical Emergency Teaching Hospital, 300041 Timisoara, Romania; 6 Bioclinica Medical Analysis Laboratory, Dreptatii Street, nr. 23, 310300 Arad, Romania

**Keywords:** vitamin D, adult population, Western Romania

## Abstract

Vitamin D, a steroid hormone synthesized primarily in the skin upon exposure to ultraviolet light, is widely deficient across global populations. This study aimed to fill the data gap in Western Romania by measuring 25-hydroxy-vitamin D levels in a cohort of 7141 from Arad County. It was observed that women, younger adults (18–29 years), and older adults (70–79 years) had notably lower vitamin D levels compared to the average population. Additionally, there was a rise in vitamin D levels over the four-year span of 2018–2022, coinciding with the COVID-19 pandemic. Our research provides fresh data on those most susceptible to vitamin D deficiency and lays the groundwork for educational campaigns on vitamin D supplementation benefits.

## 1. Introduction

Vitamin D, also known as cholecalciferol, is a steroid hormone synthesized in the skin under ultraviolet light exposure [1]. Its active metabolite, calcitriol (1,25-dihydroxycholecalciferol), plays a critical role in regulating blood levels of calcium and phosphorus, as well as in the mineralization of bone tissue [2].

According to the International Union of Pure and Applied Chemistry’s Commission on the Nomenclature of Biological Chemistry, vitamin D_3_ is classified as either a steroid or a secosteroid, with the chemical name 9,10-secocholesta-5,7,10(19)-trien-3 betaol. Among the compounds referred to as vitamin D, six different steroid hormones were described, each with varying levels of biological activity. These include cholecalciferol (D_3_), the endogenous precursor derived from cholesterol; its hydroxylated derivative, calcidiol (25(OH)D_3_), which has partial activity; and its hydroxylated derivative, the active form, calcitriol (1,25(OH)_2_D_3_). Additionally, ergocalciferol (D_2_), a plant-derived form, along with its monohydroxy- and dihydroxy metabolites, were identified [3]. Structurally, ergocalciferol differs from cholecalciferol because it has a double bond between C22 and C23 and an additional methyl group to C24 [4].

Skin synthesis has been established as the primary natural source of vitamin D production, involving the photochemical conversion of 7-dehydrocholesterol (7-DHC) into pre-vitamin D [5,6]. Vitamin D_3_ can be synthesized in this manner or, along with vitamin D_2,_ absorbed in the small intestine [7,8]. However, these forms are considered biologically inactive until they undergo enzymatic hydroxylation to become active [9].

Skin synthesis of vitamin D is triggered by exposure to ultraviolet B (UVB) radiation (280–320 nm), occurring primarily in the basal layer of the epidermis [10,11]. The photodegradation of the B ring from 7-DHC leads to the formation of pre-vitamin D, tachysterol, and lumisterol. These secondary products are converted into vitamin D. When vitamin D is released from cells, it enters the circulatory system. The transport of this vitamin to the organs occurs through vitamin D binding protein (VDBP) [12,13]. VDBP belongs to the albumin superfamily of binding proteins, which includes albumin, alpha-fetoprotein, and alpha-albumin/afamin [14,15,16]. VDBP is synthesized by hepatic parenchymal cells under the influence of estrogen, glucocorticoids, and inflammatory cytokines and plays a crucial role in binding and transporting vitamin D and its metabolites to the target organs [17,18,19]. A deficiency in VDBP can have repercussions on the functionality of vitamin D. The serum level of VDBP is significantly reduced in all conditions involving tissue necrosis or injury, such as acute liver failure, septic shock, and tissue traumatism [17].

In order to exert its hormonal activity, vitamin D undergoes two hydroxylation processes [6,20,21]. The first hydroxylation requires 25-hydroxylase, which facilitates the production of 25-hydroxyvitamin D_3_ (25(OH)D_3_) in the liver hepatocytes. 25-hydroxyvitamin currently represents the standard marker for assessing vitamin D status in humans [21,22,23,24,25]. The second hydroxylation occurs in the proximal tubes in the kidneys and is performed using 1α-hydroxylase (CYP27B1), leading to the synthesis of calcitriol (1,25(OH)_2_D_3_) [26]. The active metabolite of cholecalciferol binds to vitamin D receptors (VDR) and modulates gene expression [27]. The result is an increase in serum calcium levels through enhanced intestinal uptake of phosphorus and calcium, increased renal reabsorption of calcium, and increased osteoclast activity [28].

Vitamin D, while primarily synthesized endogenously via ultraviolet radiation exposure, can also be ingested through dietary sources and supplements. This vitamin is found in mushrooms (21.1–58.7 μg/100 g), cheese, beef liver, eggs (1.3–2.9 μg/100 g), dark chocolate (4 μg/100 g), as well as fortified foods (milk, yogurt, orange juice, and breakfast cereals) [29].

Vitamin D deficiency affects an estimated 50% of the global population, with an estimated 1 billion people worldwide belonging to different ethnicities and age groups [30]. The risk factors for this worldwide deficiency included insufficient dietary intake and decreased outdoor activities as well as environmental factors such as air pollution, which decreases the exposure to sunlight, therefore reducing the UVB-induced vitamin D synthesis [31].

Vitamin D deficiency is a significant global public health issue due to its association with all-cause mortality [32]. Insufficient levels of this vitamin are correlated with an increased risk of rickets and osteoporosis as well as chronic diseases, i.e., coronary heart disease, non-insulin-dependent diabetes, different neurological disorders, autoimmune and inflammatory diseases [31,33]. Vitamin D deficiency is widespread in the Middle East. Reporting the highest rates at 80%, Southern and Eastern Europe has a prevalence of up to 60%, while Northern Europe has a lower occurrence of less than 20% [34].

In Romania, limited data on the status of vitamin D in the adult population is available, although existing studies indicate that deficiency is prevalent [35,36]. Romania, located between 44° N and 48° N latitude in Eastern Europe, exhibits seasonal fluctuations in vitamin D levels, with peaks observed in September and the lowest level in March [37]. Currently, data on the sun protection measures adopted by the residents in Western Romania when outdoors is not documented.

Currently, Romania lacks systematic screening for vitamin D levels among its citizens. Consequently, this study was undertaken to evaluate vitamin D levels among the residents of Arad County, Western Romania.

## 2. Materials and Methods

### 2.1. Study Population

In the current study, we included 7141 consecutive residents of Arad County, Romania, from 1 January 2018 to 31 December 2021, who came in for a routine blood draw. Blood samples were collected in the Bioclinica Clinical Laboratories with blood collection points in both urban and rural regions of Arad County. The dataset included basic demographic information for each participant, such as date of birth, gender, and whether they resided in an urban or rural area. However, no clinical data were collected for this study. All patients were mobile and identified as White/Caucasian.

Participants were grouped into seven categories according to their age: 18–29 years, 30–39 years, 40–49 years, 50–59 years, 60–69 years, 70–79 years, and 80+ years.

### 2.2. Sample Collection and Laboratory Assessments

From all the study participants, venous blood samples into serum clot activator tubes were collected using the standard venipuncture techniques between 8 A.M. and 11 A.M. Due to the overnight fasting, all participants were in a fasted state. The filled clot activator tube, within an hour of collection, was then centrifuged at 2000× *g* for 10 min and then placed into Cobas 6000’s module e601 (Roche Diagnostics, Mannheim, Germany) in order to assess the level of 25-hydroxyvitamin D.

Vitamin D (25-hydroxyvitamin D) was assessed on Cobas 6000’s module e601 (Roche Diagnostics, Mannheim, Germany) using electrochemiluminescence. Levels of vitamin D above or equal to 30 µg/L were considered optimal, levels between 21 to 29 µg/L were considered insufficient, and levels below or equal to 20 µg/L were considered to be indicative of deficiency.

All determinations conducted in this study were in accordance with the manufacturer’s instructions as well as the internal laboratory standards.

### 2.3. Data Collection and Statistical Analysis

Statistical analyses were performed using Stata 16.1 (StataCorp, College Station, TX, USA). Data were presented as numbers, percentages, and mean ± standard deviation (SD).

A traffic light system (Red—vitamin D deficiency, Yellow—insufficiency, and Green—optimal) in our charts was used to enhance reader comprehension, enabling readers to quickly grasp the distribution and prevalence of vitamin D statuses within the population [38].

Descriptive statistics were used to summarize the key characteristics of the study population. Mean and standard deviation were used for continuous variables, while percentage was used for categorical variables. Ordinal logistic regression was employed as the primary statistical model for this study, chosen for its appropriateness in analyzing ordinal outcome variables. The model facilitated the exploration of associations between vitamin D levels categorized as insufficient, deficient, and optimal and various independent variables. Odds ratios (OR) with their corresponding 95% confidence intervals (95% CI) were presented for each statistical analysis. Statistical significance for both logistic regression models was set at *p* < 0.05.

### 2.4. Ethical Approval

This study was approved by the “Vasile Goldis” University Ethics Committee, Arad, Romania. (no. 15 from 31 March 2023).

## 3. Results

### 3.1. Descriptive Statistics of the Participants Stratified by Age, Gender, Area of Residence, and Year of Blood Collection

A total of 7141 adult participants from Arad County, Western Romania, were enrolled in this study. They were aged between 18 and 97 years (mean = 48.66, median = 48).

**Figure 1:**
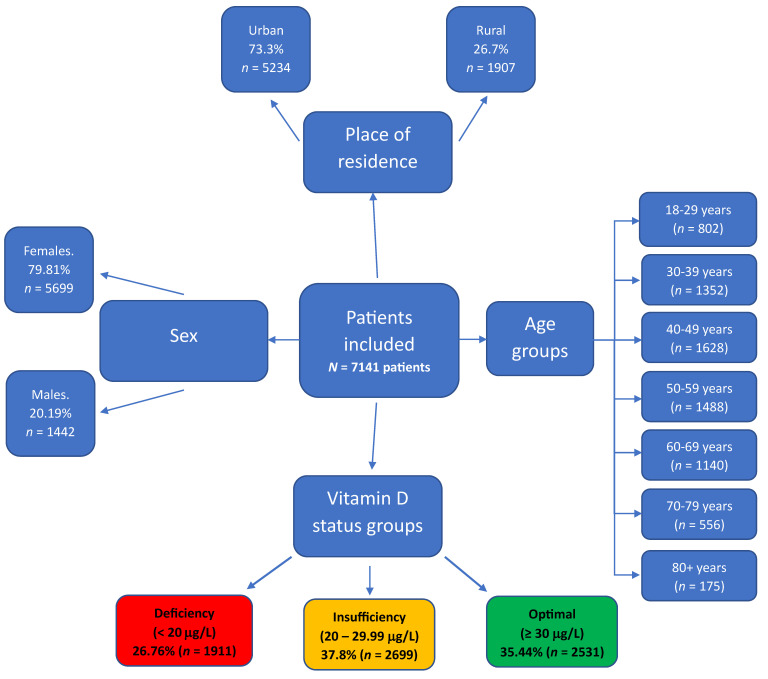
Comprehensive diagram depicting the distribution of 7141 study participants from Arad County, Western Romania, by sex, area of residence, age groups, and vitamin D status.

When data was stratified by sex and area of residence, it was found that out of the total participants, 5699 (79.81%) were female, and 5234 (73.3%) were residing in urban areas. Age-wise distribution was as follows: 802 patients (11.23%) were aged 18–29 years, 1352 (18.92%) were 30–39 years, 1628 (22.80%) were 40–49 years, 1488 (20.82%) were between 50–59 years, 1140 (15.97%) were between 60–65 years, 556 (7.78%) were 70–79 years, and 175 participants (2.45%) were over 80 years (Figure 1).

### 3.2. Descriptive Analysis and Ordinal Logistic Regression Models Analysing Vitamin D Levels according to Participant’s Sex and Area of Residence

Of the 7141 study participants, 1911 (26.76%) had vitamin D deficiency, 2688 (37.8%) had insufficient levels of vitamin D, and 2531 (35.44%) had optimal vitamin D levels. Among the male participants (*n* = 1442), 315 (21.84%) had vitamin D deficiency, 549 (38.08%) had insufficient levels, and 578 (40.08%) maintained optimal levels of vitamin D. Within the female cohort (*n* = 5699), 1596 (28%) had deficiency, 2150 (37.73%) had insufficiency, and 1953 (34.27%) had optimal levels of vitamin D.

Within the subgroup of participants residing in rural areas (*n* = 1907), 494 (25.9%) had deficiency, 784 (41.11%) had insufficiency, and 629 (32.99%) had optimal levels of vitamin D. For those residing in urban areas (*n* = 5234), 1417 (27.07%) had deficiency, 1915 (36.59%) had insufficiency, and 1902 (36.34%) had optimal levels of vitamin D (Table 1).

After performing ordinal logistic regression on the sex variable, it was revealed that females were generally less likely to have higher vitamin levels compared to males (OR = −0.28, 95% CI: −0.39–−0.17, *p* ≤ 0.001).

When comparing rural and urban residents, we observed that those living in urban areas have a slightly higher percentage of vitamin deficiency than those in rural areas. However, urban residents also had a slightly higher percentage in the “optimal” vitamin D level category, although not reaching statistical significance (OR = 0.06, 95% CI: −0.04–0.15, *p* value = 0.244) (Table 1).

### 3.3. Descriptive Analysis and Ordinal Logistic Regression Models Analysing Vitamin D Levels according to Participant’s Age at the Time of the Blood Draw

In the age group of 18–29 years, 32.29% of individuals had deficiency (<20 ng/dl) of vitamin D, 39.65% exhibited insufficiency (20–30 ng/dl), and 28.06% had optimal (>30 ng/dl) levels. For those aged 30–39 years, 25.81% had a deficiency, 40.83% showed insufficiency, and 33.36% demonstrated optimal vitamin D levels. In the 40–49 age group, 26.23% experienced deficiency, 40.23% had insufficiency, and 33.54% achieved optimal vitamin D levels. Within the age range of 50–59 years, 24.19% had a deficiency, 35.69% displayed insufficiency, and 40.12% had optimal vitamin D levels. Among individuals aged 60–69 years, 24.74% had a deficiency, 35.61% exhibited in the 70–79 age group, 32.19% had a deficiency, 34.17% showed insufficiency, and 33.64% had optimal vitamin D levels. Among those aged 80 years and older, 31.43% had a deficiency, 26.86% experienced insufficiency, and 41.71% had optimal vitamin D levels (Table 2).

In a ordinal logistic regression model with the young adults group (aged between 18 and 29 years) as the reference, a statistically significant increased likelihood of vitamin D insufficiency or deficiency was found in comparison to the 30–39 years age group (OR = 0.27, 95% CI = 0.11–0.43, *p* < 0.001), 40–49 years (OR = 0.27, 95% CI = 0.11–0.42, *p* < 0.001), 50–59 years (OR = 0.48, 95% CI = 0.32–0.64, *p* < 0.001), 60–69 years (OR = 0.45, 95% CI = 0.29–0.62, *p* < 0.001), and 80+ years (OR = 0.36, 95% CI = 0.05–0.67, *p* = 0.02). However, there was no statistically significant difference when comparing young adults to those aged between 70 and 79 years old (OR = 0.13, 95% CI = −0.07–0.33, *p* = 0.19) (Table 2).

### 3.4. Descriptive Analysis and Ordinal Logistic Regression Models Analysing Vitamin D Levels according to the Year of the Blood Draw

In 2018, of 1414 participants, 33.52% had a deficiency, 37.63% exhibited insufficiency, and 28.85% achieved optimal levels. Similarly, in 2019, 28.14% exhibited deficiency, 38.18% had insufficiency, and 33.68% achieved optimal levels among 2022 participants. The year 2020 saw 22.39% with deficiency, 38.58% with insufficiency, and 39.03% reaching optimal levels among 2224 participants. Finally, in 2021, 24.98% had a deficiency, 36.26% exhibited insufficiency, and 38.76% reached optimal levels among 1481 participants. These findings provide insights into the changing trends of vitamin D status in the study population over the four-year period (Table 3).

When the year 2018 was used as a reference in the ordinal logistic regression model and compared to 2019 (OR = 1.24, 95% CI = 0.11–0.37, *p* < 0.001), 2020 (OR = 1.50, 95% CI = 0.38–0.62, *p* < 0.001), and 2021 (OR = 1.44, 95% CI = 0.3–0.57, *p* < 0.001), a statistically significant increase in the overall levels of vitamin D was observed over the years (Table 3).

## 4. Discussion

Vitamin D deficiency is recognized as a significant public health issue worldwide, affecting roughly one billion people, with an estimated half of the global population presumed to be deficient [30,39]. This deficiency is notably prevalent in Middle Eastern countries, particularly among individuals with a higher amount of melanin in their skin and those who traditionally cover most of their skin. For instance, in the case of infants in India, Iran, and Turkey, over 90% of them were deficient in vitamin D. In the United States, vitamin D deficiency was present in 47% of African-American infants and 56% of Caucasian infants. In terms of the prevalence of vitamin D deficiency in the adult population, over 80% of adults in Pakistan, India, and Bangladesh were deficient, while in the United States, vitamin D deficiency was present in 35% of adults [39]. In Western, Southern, and Eastern Europe, the prevalence of vitamin D deficiency was between 30 and 60%, while in Northern Europe, it exceeded 20% [34].

This study revealed that out of 7141 study participants, 1911 (26.76%) had a deficiency in vitamin D levels, 2688 (37.8%) had insufficient levels, and 2531 (35.44%) had optimal levels. These findings align closely with those of Bucurica et al. [40], who assessed the vitamin D status of hospitalized patients in Romania over a two-year period. In this study, which involved 11,182 participants, 28.83% were found to have vitamin D deficiency, 32.11% had insufficient levels, and 39.05% had optimal vitamin D levels [40].

Our study reported that females had an increased likelihood of vitamin D deficiency compared to males, aligning with the findings of Muscogiuri et al. [41]. Their research, involving 500 adult Caucasians in Naples, Italy, found that vitamin D levels were significantly higher in males despite similar sun exposure and a lack of vitamin D supplements among all participants [41]. Our results are also consistent with Chirita-Emandi et al. [35], who reported a higher risk for vitamin D deficiency in females across a sample of 6.631 individuals. Further supporting these gender differences, da Silveira et al.’s [42] study revealed that females of childbearing age from Brazil had a high deficiency of vitamin D [42]. Contributing factors were found to be marital status [43] and lower socioeconomic status [44]. In contrast, the Qatar Biobank research by Al-Dabhani et al. [45] reported a higher prevalence of vitamin D deficiency in males (69%) compared to females (61%), with females actually showing higher serum 25(OH)D concentrations [45].

Our research indicated that individuals aged 70–79 years generally exhibit lower vitamin D levels compared to the younger adult population. This observation is in line with the findings reported by Giustina et al. [46], who noted that reduced sun exposure and a decline in the skin’s ability to produce vitamin D put older adults at risk for deficiency. The study emphasizes the importance of maintaining adequate vitamin D levels to prevent various health issues, including bone density reduction, osteomalacia, fractures, and other potential extra-skeletal effects like diabetes and cardiovascular disease, affecting mainly the older population. The consensus is that vitamin D supplementation combined with calcium is beneficial for reducing fracture risks in the elderly [46]. Similar results were also reported by Bucurica et al. [40] and Chirita-Emandi et al. [35] in Romania. In elderly persons, the prevalence of vitamin D deficiency was 96% in India, 90% in Turkey, 72% in Pakistan, 67% in Iran, and 61% in the United States [47]. The increased incidence of deficiency in older adults is also attributed to a reduced capacity for cutaneous vitamin D synthesis and less sunlight exposure [48]. Assessing vitamin D levels in older individuals is essential due to the role that this vitamin plays in the regulation of calcium–phosphorus homeostasis, which contributes to the maintenance of bone health [20,49].

Romania, located between 44° N and 48° N latitude, shows parallels to findings in France, located approximately from 43.5° N to 48° N, where a third of healthy adults were reported to be vitamin D deficient [50]. Similar results were also reported in Spain (located between approximately 36° N and 43.5° N latitude) by González-Molero et al., who found that 33.9% of the Spanish population is at risk for vitamin D deficiency [51]. Significantly higher rates of vitamin D deficiency (57.2% of the studied participants) were reported by Capuano et al. [52] in Italy (located between latitudes 35° and 47° N). Controversially, in Ukraine (located approximately 44° N to 52° N latitude), vitamin D deficiency was reported at a lower rate of 19.5%, which is less than the rate observed in our study [53].

We also reported that younger adults are more likely to experience deficient or insufficient vitamin D levels. This aligns with the findings of Tangpricha et al. [54], who reported a 36% vitamin D deficiency in adults aged 18–29 from the USA. A possible explanation is that young adults tend to have lower consumption of vitamin D-containing foods such as fortified cereals and oily fish [55], also due to the seasonal variation in sunlight exposure, especially during the winter [54]. Besides dietary intake and sun exposure habits, young adults aged 18–29 often spend increased time indoors due to their attendance in college or graduate school, contributing to a greater prevalence of vitamin D deficiency within this demographic, as documented by multiple studies [56,57,58].

Our results revealed that study participants aged 80+ had the highest percentage (41.71%) of optimal vitamin D levels. This may be attributed to their risk of fractures [59] and the potential fatal outcome of a fracture at advanced ages [60]. Furthermore, adherence to COVID-19 guidelines, which recommend vitamin D supplementation, was higher among the elderly [61,62]. To better understand this trend, it is recommended that future research in this area should include specific questionnaires.

The current study revealed that levels of vitamin D in the adult population tended to increase as the pandemic began and progressed. The SARS-CoV-2 pandemic began in December 2019 when an outbreak of severe pneumonia of unknown cause was identified in Wuhan, China [63]. Romania confirmed its first case of SARS-CoV-2 on 26 February 2020 [64]. A plausible rationale for this rise in vitamin D levels during this period may be attributed to increased public health recommendations for self-isolation [65], which likely prompted a rise in the consumption of vitamin D supplements as a preventive health measure [66].

Several studies highlighted a correlation between low vitamin D levels and increased susceptibility to acute respiratory infections, including SARS-CoV-2 [67]. Additionally, higher levels of vitamin D have been associated with a lower risk of ICU hospitalization in patients with COVID-19 [68,69]. Specifically concerning COVID-19, a study conducted by Abdrabbo et al. [70] highlighted the interaction between the SARS-CoV-2 spike protein and the human angiotensin-converting enzyme 2, which is influenced by the disulfide-thiol balance in host cells. Vitamin D supplementation may reduce oxidative stress, affecting the host cell redox status. This could potentially block viral entry and prevent or reduce the severity of COVID-19 infection. However, the precise molecular mechanisms of this interplay remain unclear and require further research [70].

Maintaining adequate vitamin D levels is crucial for the general population due to its immunostimulatory and immunomodulatory effects [71]. The significance of vitamin D has expanded beyond bone and mineral metabolism as the vitamin D receptor (VDR) and the enzyme responsible for its activation, 1-α-hydroxylase (CYP27B1), are expressed in various types of cells, including those in the pancreas, intestine, and immune system cells [72,73].

Vitamin D plays a role in regulating innate immunity by enhancing the body’s defense mechanism against microbes and other pathogenic organisms, as well as in modulating the adaptive immune system through its direct effects on T-cell activation and the phenotype and function of antigen-presenting cells, especially dendritic cells [71]. The pivotal role of vitamin D in immune function was first understood through the discovery of the expression of the vitamin D receptor (VDR) expression across nearly all cells of the immune system [74]. VDR belongs to the superfamily of nuclear receptors [75] and acts on several genes in about half of human cells and tissues [76]. 1,25-(OH)_2_D_3_ binds to the VDR, determining a conformational rearrangement of the molecule that will allow its heterodimerization with the retinoid X receptor (RXR). Subsequently, this complex is translocated into the nucleus, where it binds to vitamin D response elements (VDRES) in the promoter region of the target genes and where it modulates their transcription [77]. The VDR–RXR complex can regulate the expression of more than 3000 genes in the human genome, depending on the cell type and physiological conditions [78,79,80]. Therefore, 1,25-(OH)_2_D_3_ regulates numerous cellular processes by activating the nuclear receptor VDR [81]. VDR is expressed differently in immune cells depending on their activation state. For example, at the time of activation, T cells present a higher concentration of VDR with an increase that is significant after eight hours and reaches a maximum level 48 h after activation [82]. In contrast, monocytes lose the expression of VDRs by differentiating them into macrophages or dendritic cells [83].

Our study acknowledges certain limitations. A notably higher number of females and urban residents were included in this study, which may reflect a tendency of these groups to access healthcare services and undergo health assessments more frequently [35,84]. An additional constraint was the absence of a questionnaire to collect data on participants‘ associated health conditions, body mass index (BMI), intake of supplements, or dietary habits. Furthermore, the lack of precise timing for blood sample collection means the data could not be sorted according to seasons of specific months, which may influence vitamin D levels. Lastly, another limitation was that the adult population does not represent the broader general population but, due to the high number of study participants, could offer insights into the current trends within the general population.

## 5. Conclusions

The results of our research show a notable occurrence of below-optimal vitamin D levels within the adult population of Western Romania. A heightened risk of deficiency was particularly evident in females, as well across age extremes, affecting both the young and elderly. In addition, there was a discernible increase in vitamin D levels among the adult population over a four-year interval during the COVID-19 pandemic. This trend emphasizes the critical need for widespread education and communication regarding the importance of vitamin D supplementation, given the significant role of vitamin D in immune function.

## Figures and Tables

**Table 1:** Ordinal logistic regression for two variables (sex and area of residence) on vitamin D status among study participants from Arad County, Western Romania.

Sex (*n* = 100%)	Deficiency (<20 µg/L)	Insufficiency (20–29.9 µg/L)	Optimal (>30 µg/L)	OR	95% CI (Min–Max)	*p* Value
Male (*n* = 1442)	315 (21.84%)	549 (38.08%)	578 (40.08%)	Ref.		
Female (*n* = 5699)	1596 (28%)	2150 (37.73%)	1953 (34.27%)	−0.28	−0.39–−0.17	<0.001
**Area of residence**						
Rural (*n* = 1907)	494 (25.9%)	784 (41.11%)	629 (32.99%)	Ref.		
Urban (*n* = 5234)	1417 (27.07%)	1915 (36.59%)	1902 (36.34%)	0.06	−0.04–0.15	0.244

**Table 2:** Analysis of vitamin D status across age groups in study participants from Arad County, Western Romania.

Age Group (*n* = 100%)	Deficiency (<20 µg/L)	Insufficiency (20–29.9 µg/L)	Optimal (>30 µg/L)	OR	95% CI (Min–Max)	*p* Value
18–29 years (*n* = 802)	259 (32.29%)	318 (39.65%)	225 (28.06%)	Ref.		
30–39 years (*n* = 1352)	349 (25.81%)	552 (40.83%)	451 (33.36%)	0.27	0.11–0.43	<0.001
40–49 years (*n* = 1628)	427 (26.23%)	655 (40.23%)	546 (33.54%)	0.27	0.11–0.42	<0.001
50–59 years (*n* = 1488)	360 (24.19%)	531 (35.69%)	597 (40.12%)	0.48	0.32–0.64	<0.001
60–69 years (*n* = 1140)	282 (24.74%)	406 (35.61%)	452 (39.65%)	0.45	0.29–0.62	<0.001
70–79 years (*n* = 556)	179 (32.19%)	190 (34.17%)	187 (33.64%)	0.13	−0.07–0.33	0.19
80+ years (*n* = 175)	55 (31.43%)	47 (26.86%)	73 (41.71%)	0.36	0.05–0.67	0.02

**Table 3:** Analysis of vitamin D status distribution trends over four consecutive years (2018–2021) among study participants from Arad County, Western Romania.

Year (*n* = 100%)	Deficiency (<20 µg/L)	Insufficiency (20–29.9 µg/L)	Optimal (>30 µg/L)	Adjusted OR	95% CI (Min–Max)	*p* Value
2018 (*n* = 1414)	474 (33.52%)	532 (37.63%)	408 (28.85%)	Ref.		
2019 (*n* = 2022)	569 (28.14%)	772 (38.18%)	681 (33.68%)	0.24	0.11–0.37	<0.001
2020 (*n* = 2224)	498 (22.39%)	858 (38.58%)	868 (39.03%)	0.50	0.38–0.62	<0.001
2021 (*n* = 1481)	370 (24.98%)	537 (36.26%)	574 (38.76%)	0.44	0.3–0.57	<0.001

## Data Availability

Datasets used and/or analyzed during the current study are available from the corresponding author on reasonable request.
